# Anorexia Nervosa as a Metabo-Psychiatric Disorder: Consequences for Assessment and Treatment in Childhood and Adolescence

**DOI:** 10.1192/j.eurpsy.2022.56

**Published:** 2022-09-01

**Authors:** B. Herpertz-Dahlmann, J. Seitz, B. Dahmen

**Affiliations:** RWTH Aachen, Department Of Child And Adolescent Psychiatry, Psychosomatics And Psychotherapy, Aachen, Germany

## Abstract

Anorexia Nervosa (AN) has the highest mortality of all psychiatric disorders. Patients who experience the onset of AN in their teens suffer on average 10 years from an eating or other mental disorder. Recently many clinicians in the US and Europe have addressed the inadequacy of treatment for this disabling disease which – at least in adult patients - is mainly based on individual psychotherapeutic interventions. Against the background of a recent GWAS and genetic correlations with other mental disorders, but also inverse correlations with important metabolic parameters such as fasting insulin and leptin new treatment strategies must be developed. This presentation wants to elucidate two different interventions, a biological strategy, and a more pragmatic treatment setting in the “real world”. The biological strategy refers to the gut-brain axis which has been shown to be severely affected in AN. Patients show a deep perturbation of the gut microbiome with no significant improvement with weight rehabilitation. Thus, additional ways must be found such as nutritional strategies (e.g., administration of pre- or probiotics, stool transfer) to probably improve the outcome of this disorder. In addition, inpatient treatment which is still the gold standard for treatment of AN in Europe, has not been proven to alter the long-term prognosis of AN. Consequently, other interventions such as home treatment which involve the family and interrupt eating disorder associated habits as disease preserving factors will be introduced and the results of a pilot study in adolescent AN be presented.

**Disclosure:**

European Commission/ERA NET Research Grant Ministry of Labour, Health and Social Policies of Research Grant North-Rhine-Westfalia, Germany Joint Federal Committee (GBA) Rese

